# Personal

**Published:** 1891-06

**Authors:** 


### Personal.

Dr. H. O. Walker, of Detroit, Mich., has been appointed surgeon to the police department in that city, vice Dr. J. B. Book, resigned. Dr. Walker is an eminent surgeon and one of Detroit's most prominent physicians. He brings to his new position not only the skill of an experienced surgeon, but an executive capacity worthy the important trust. The city is to be congratulated that can obtain the professional services of such a man.

Removal.—Dr. G. W. McCray, the well-known druggist and manufacturing pharmacist, who was located for so many years at 213 Main street, Buffalo, has removed to 897 Main street. This is one of the best up-town locations in the new "Strathmore," and Dr. McCray is to be congratulated upon a progressive and enterprising move.

The appointment of Dr. E. C. W. O'Brien to the position of Fire Surgeon, which has been announced, is a fitting recognition of his gratuitous services to members of that department for a number of years. The choice is a good one.

Dr. M. B. Folwell sailed, May 16th, for Europe, accompanied by his family. He will return the latter part of August.

